# Supplementary material for: Novel Driver Strength Index highlights important cancer genes in TCGA PanCanAtlas patients
Source: PeerJ. 2022 Aug 11;10:e13860. doi: 10.7717/peerj.13860 (PMC9375969; doi:10.7717/peerj.13860)
Supplement: Supplemental Information 2 [file peerj-10-13860-s002.zip › COHORTS/genes plots/detailed/2021_8_16_14_9_distribution_events_detailed_females_3.pdf]

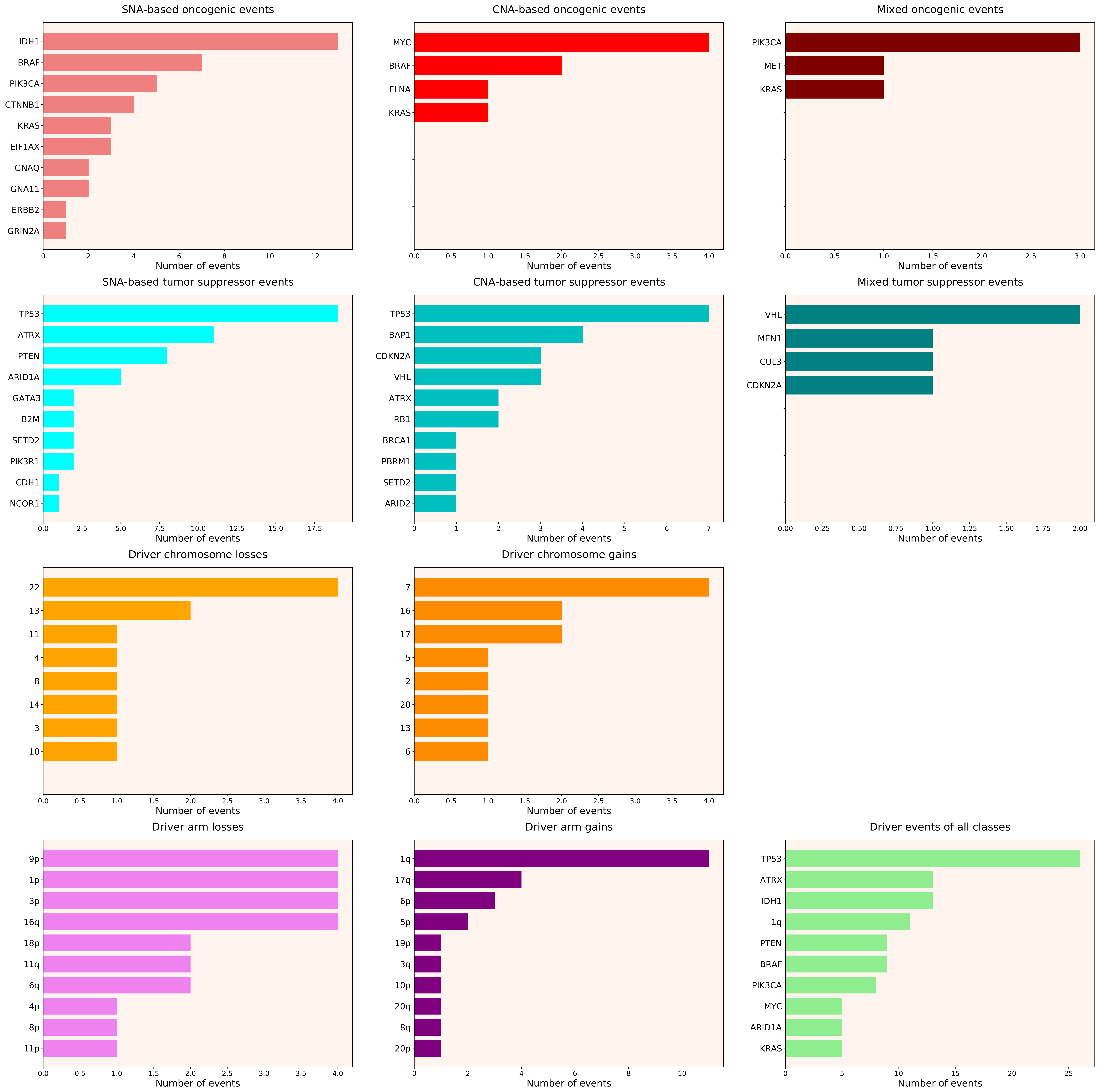
SNA-based oncogenic events
IDH1
BRAF
PIK3CA
CTNNB1
KRAS
EIF1AX
GNAQ
GNA11
ERBB2
GRIN2A
Number of events
CNA-based oncogenic events
MYC
BRAF
FLNA
KRAS
Number of events
Mixed oncogenic events
PIK3CA
MET
KRAS
Number of events
SNA-based tumor suppressor events
TP53
ATRX
PTEN
ARID1A
GATA3
B2M
SETD2
PIK3R1
CDH1
NCOR1
Number of events
CNA-based tumor suppressor events
TP53
BAP1
CDKN2A
VHL
ATRX
RB1
BRCA1
PBRM1
SETD2
ARID2
Number of events
Mixed tumor suppressor events
VHL
MEN1
CUL3
CDKN2A
Number of events
Driver chromosome losses
22
13
11
4
8
14
3
10
Number of events
Driver chromosome gains
7
16
17
5
2
20
13
6
Number of events
Driver arm losses
9p
1p
3p
16q
18p
11q
6q
4p
8p
11p
Number of events
Driver arm gains
1q
17q
6p
5p
19p
3q
10p
20q
8q
20p
Number of events
Driver events of all classes
TP53
ATRX
IDH1
1q
PTEN
BRAF
PIK3CA
MYC
ARID1A
KRAS
Number of events
